# Effect of PI3K/Akt Signaling Pathway on PRAS40Thr246 Phosphorylation in Gastric Cancer Cells

**Published:** 2019-12

**Authors:** Yizhuo LU, Lianghui LI, Guoyang WU, Huiqin ZHUO, Guoyan LIU, Jianchun CAI

**Affiliations:** 1. Department of General Surgery, Zhongshan Hospital, Xiamen University, Xiamen, P.R. China; 2. Institute of Gastrointestinal Oncology, School of Medcine, Xiamen University, Xiamen, P.R. China; 3. Department of Gastrointestinal Surgery, Zhongshan Hospital, Xiamen University, Xiamen, P.R. China

**Keywords:** Gastric cancer, LY294002, MK-2206, Phosphorylation, PI3K/Akt signaling pathway, PRAS40-Thr246

## Abstract

**Background:**

We aimed to investigate the effect of PI3K/Akt signaling pathway on PRAS40Thr246 phosphorylation in gastric cancer cells.

**Methods:**

The study was conducted from April 2017 to January 2018 in Zhongshan Hospital, Xiamen University, Xiamen, China. Gastric cancer cells were divided into three groups: gastric cancer cell group, LY294002 group and MK-2206 group. Specific tests were conducted accordingly.

**Results:**

Inhibition of PI3K/Akt signaling pathway activation and PRAS40Thr246 phosphorylation could inhibit proliferation and invasion and promote apoptosis of gastric cancer cells, and PRAS40Thr246 phosphorylation could activate PI3K/Akt signaling pathway.

**Conclusion:**

The levels of PI3K/Akt signaling pathway related proteins and p-PRAS40Thr246 were significantly increased in gastric cancer cells. p-PRAS40-Thr246 was able to reflect the activation of the PI3K/Akt signaling pathway, reflecting the sensitivity of the PI3K/AKT signaling pathway to inhibitors.

## Introduction

Gastric cancer is the fourth most common cancer and the second leading cause of cancer-related deaths in the world, accounting for 6.8% of all new cancer cases worldwide. In addition, it has a poor prognosis, only 5% of patients with gastric cancer can survive at least 5 years after diagnosis, and the mortality accounts for 10% of all cancer cases (1, 2). Although progress has been made in understanding the risk factors associated with gastric cancer, the etiology is not entirely clear. It is widely believed that progressive changes of multiple molecules cause the development of gastric cancer (3, 4). Therefore, it is very important to find molecular markers that can be used as prognostic factors for effective treatment of gastric cancer.

PI3K/AKT pathway is the main signal pathway of downstream of many growth factor receptors and the most active signal pathway in human tumors. It also promotes the proliferation and malignant transformation of tumor cells through phosphorylation of PI3K and AKT protein and inhibits the apoptosis of tumor cells. Therefore, PI3K/AKT inhibitors have been widely used in cancer therapy (5, 6). Many studies have reported the role of PI3K/AKT pathway in promoting the proliferation and invasion of gastric cancer, but there has been no consensus on the precise definition of molecular markers of PI3K/AKT pathway activation (7, 8). PRAS40 is a substrate for protein kinase B.

The level of Thr246 phosphorylation of PRAS40 (p-PRAS40-Thr246) can predict the sensitivity of prostate cancer cells and triple-negative breast cancer cells against AKT inhibitors. The clinical application of PI3K/AKT inhibitors, and can accurately indicate the activation state of PI3K/AKT pathway (9). Moreover, p-PRAS40-Thr246 was associated with malignant progression and poor prognosis of gastric cancer (10).

However, there are few reports of p-PRAS40-Thr246 and PI3K/AKT pathway in gastric cancer, and there are no studies on whether PI3K/AKT inhibitors affect p-PRAS40-Thr246 phosphorylation in gastric cancer cells. Therefore, this study was conducted on these issues.

## Methods

### Cell sources

Human gastric carcinoma cells were purchased from Shanghai Youduo Biotechnology Co., Ltd., cat no. KG189-G; Human normal gastric mucosal epithelial cells were purchased from Shanghai Zishi Biotechnology Co., Ltd., cat no. wa100527. The culture mediums were DMEM medium (Xiamen Yanke Biotechnology Co., Ltd., cat no. 10567014) containing 10% fetal calf serum (Beijing Think-far Technology Co., Ltd., cat no. 10099141), with the culture conditions of 37 °C, 5% CO_2_ and 90% humidity.

### Experimental grouping

The study was conducted from April 2017 to January 2018 in Zhongshan Hospital. Gastric mucosal epithelial cells and gastric cancer cells were routinely subcultured after rapidly thawing in a 37°C water bath + resuscitation in complete culture solution for 5–8 hours, and after passage, gastric cancer cells were divided into three groups: gastric cancer cell group, LY294002 group and MK-2206 group. Cells in the LY294002 group were treated with 20 um of PI3K inhibitor LY294002 (TargetMol, China, cat no. T2008); cells in the MK-2206 group were treated with 4 nmol/L of AKT inhibitor MK-2206 (Wuhan Booute Biotechnology Co.,Ltd., cat no. orb322834), and the cells in the gastric cancer cell group were not treated. The levels of PI3K, Akt, phosphorylated PI3K (p-PI3K), phosphorylated Akt (p-Akt) and p-PRAS40-Thr246 in the four groups were measured, and the cell proliferation, invasion and apoptosis in the gastric cancer cell group, LY294002 group and MK-2206 group were detected.

### Western blot

Extraction of proteins from the four groups of cells by repeated freeze-thaw, then the proteins were separated by polyacrylamide gel electrophoresis (Beijing Dingguo Changsheng co., Ltd), with an initial voltage of 90 V. After completion of the electrophoresis, transmembrane was performed immediately at 100 V constant voltage for 100 min, then the membrane sealing solution was added at 37 °C for 60 min. Then antigen-antibody hybridization was carried out. The membrane was incubated overnight with the first antibody at 4 °C, rinsed 3 times with PBS on the next day, 10 min/time, then incubated with horseradish peroxidase labeled second antibody at 37 °C for 2 h. Chemiluminescence substrate was added to develop and fix after completion, and the film scanned bands were analyzed by SYNGENE ChemGenius imaging system. Protein relative expression level=band gray value/internal parameter gray value. PI3K, Akt, p-PI3K, p-Akt, p-PRAS40-Thr246 monoclonal antibodies and rabbit anti-human polyclonal antibodies were purchased from Abcam, USA, and Western blot test kit was purchased from Shanghai Beyotime Tian Company.

### MTT cell proliferation test

Cells in the gastric cancer cell group, LY294002 group and MK-2206 group were inoculated into 96-well plate after routine digestion, about 4,000 cells per well. MTT reagent (5 mg/mL) was added after inoculation at 37 °C for 24 h, 48 h, 72 h, 96 h and 120 h, 20 μL per well. After culturing at 37°Cfor 4 h, the supernatant containing impurities was removed, and dimethylsulfoxide was added, and then the plate was placed on the horizontal shaking table for 10 min. The absorbance values of each well were measured at 570 nm, 6 parallel holes per time point. MTT kit was purchased from sigma Company, USA.

### Cell apoptosis test

Cells in the gastric cancer cell group, LY294002 group and MK-2206 group were treated with trypsin (0.25%)-EDTA and centrifuged at 1000 rpm and at room temperature for 5 min. AnnexinV-FITC was used to label cells for 20 min, thus to detect the apoptosis rate. Life flow cytometry was purchased from Jiangsu Bomeida Life Science Co., Ltd., and AnnexinV-FITC was purchased from Beijing Luyuan Bode Biotechnology Co., Ltd., cat no. 130-097-928.

### Transwell cell invasion test

The Transwell chamber was precoated with Matrigel, and the gastric cancer cells were prepared into a single cell suspension of 1×10^5^/mL/mL and inoculated with 100μL in the Transwell chamber. Total of 100ml of DMEM medium+10% calf serum+ cell suspension was added in the upper chamber; total of 500 μL of DMEM medium+20% calf serum was added in the lower chamber. After culturing at 37 °C, 5% CO_2_ for 48 h, the cells were stained and the perforating cell number was measured. A total of 6 chambers were counted, with 6 visual fields per chamber and 3 parallel trials simultaneously. Transwell chamber and related reagents were purchased from Shanghai Yuanzi Biotechnology Co., Ltd.

### Statistical methods

SPSS19.0 (Asia Analytics Formerly Spss China) was used for the statistical analysis. The counting data were expressed as [n (%)], and the rate was compared by χ^2^ test. The measurement data were expressed as (x̄±sd), and the comparison between the two groups was performed by *t* test. Muti-group comparison and subsequent pairwise comparison were tested by univariate ANOVA combined with post Bonferonni. Comparison of different time within groups was performed by repeated variance measurement. The correlation between p-PRAS40-Thr246 and p-PI3K, p-AKT was analyzed by Pearson. *P*< 0.05 had statistical significance.

## Results

**Fig. 1:**
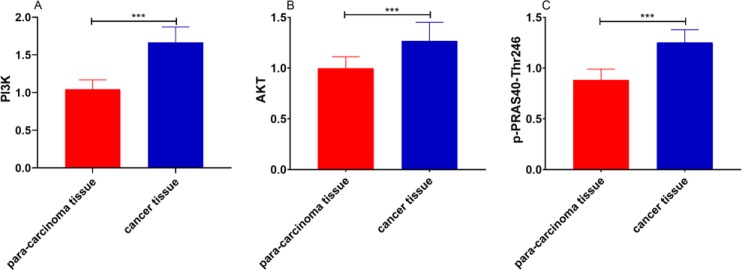
Expressions of PI3K/Akt Pathway-related proteins and p-PRAS40-Thr246 in gastric cancer tissue **A.** Expressions of PI3K in gastric cancer tissue. **B.** Expressions of AKT in gastric cancer tissue. **C.** Expressions of p-PRAS40-Thr246 in gastric cancer tissue. *** represents *P*<0.001

The levels of PI3K, AKT, and p-PRAS40-Thr246 in gastric cancer tissue were higher than those in adjacent tissue (*P*<0.001) (Fig. 1).

**Fig. 2:**
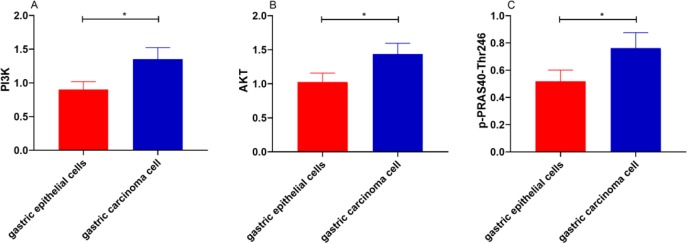
Expressions of PI3K/Akt Pathway-related proteins and p-PRAS40-Thr246 in gastric cancer cells **A.** Expressions of PI3K in gastric cancer cells. **B.** Expressions of AKT in gastric cancer cells. **C.** Expressions of p-PRAS40-Thr246 in gastric cancer cells. * represents *P*<0.05

The levels of PI3K (*P*=0.020), AKT (*P*=0.026), p-PRAS40-Thr246 (*P*=0.040) in gastric cancer cells were higher than those in gastric mucosal epithelial cells (Fig. 2).

**Fig. 3:**
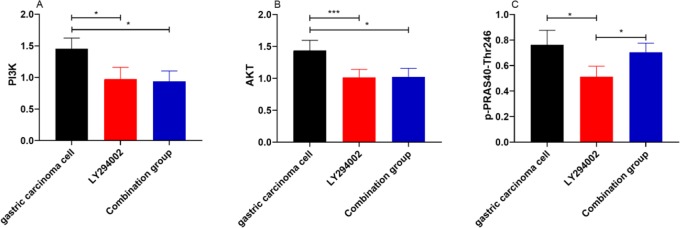
Expressions of PI3K/Akt Pathway-related proteins and p-PRAS40-Thr246 in gastric cancer cells after intervention **A.** Expressions of PI3K in gastric cancer cells. **B.** Expressions of AKT in gastric cancer cells. **C.** Expressions of p-PRAS40-Thr246 in gastric cancer cells. * represents *P*<0.05; *** represents *P*<0.001

There were significant differences in the levels of PI3K (*p*=0.019), ATK (*P*=0.016) protein and p-PRAS40-Thr246 (*P*=0.035) between the gastric cancer cell group, LY294002 group and combination group. The levels of PI3K (*P*=0.015), ATK (*P*=0.010) and p-PRAS40-Thr246 (*P*=0.015) in the LY294002 group were lower than those in the gastric cancer cell group. The levels of PI3K (*P*=0.011) and ATK (*P*=0.011) in the combination group were lower than those in the gastric cancer cell group, and there was no significant difference in the level of p-PRAS40-Thr246 between the combination group and the gastric cancer cell group. There was no significant difference in the levels of PI3K and ATK between the LY294002 group and the combination group. The level of p-PRAS40-Thr246 in the LY294002 group was lower than that in the gastric cancer cell group (*P*=0.041) (Fig. 3).

### Correlation analysis

**Fig. 4:**
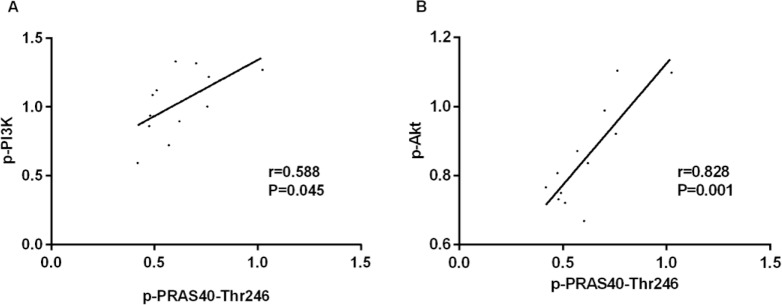
Pearson correlation analysis of p-PRAS40-Thr246 with PI3K and AKT. **A.** p-PRAS40-Thr246 was positively related with p-PI3K **B.** p-PRAS40-Thr246 was positively related with p-AKT

The levels of p-PRAS40-Thr246, PI3K and AKT in gastric cancer cells of the three groups were all included in the Pearson correlation analysis, and the results showed that p-PRAS40-Thr246 was positively related with PI3K (r=0.588, *P*=0.045), AKT (r=0.828, *P*=0.001) (Fig. 4).

### The proliferation of cells in three groups

**Fig. 5:**
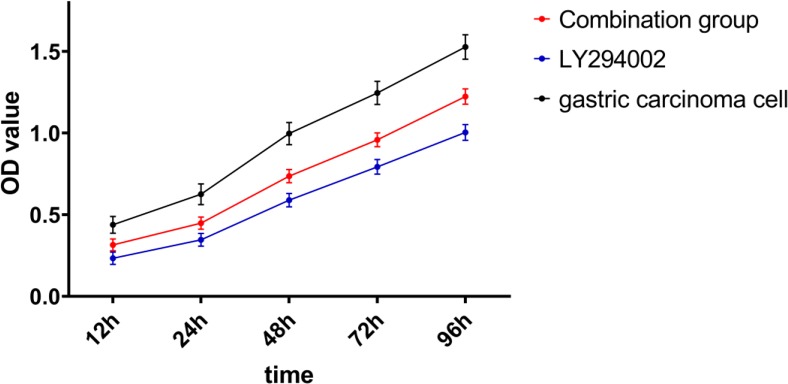
The proliferation of cells in three groups The absorbance values of cells in the LY294002 group and MK-2206 group

The absorbance values of cells in the LY294002 group and combination group were lower than those in the gastric cancer cell group after 12 h, 24 h, 48 h, 72 h, 96 h (*P*<0.001), and it was higher in the combination group than in the LY294002 group (*P*<0.001) (Fig. 5).

### The apoptosis of cells in three groups

**Fig. 6:**
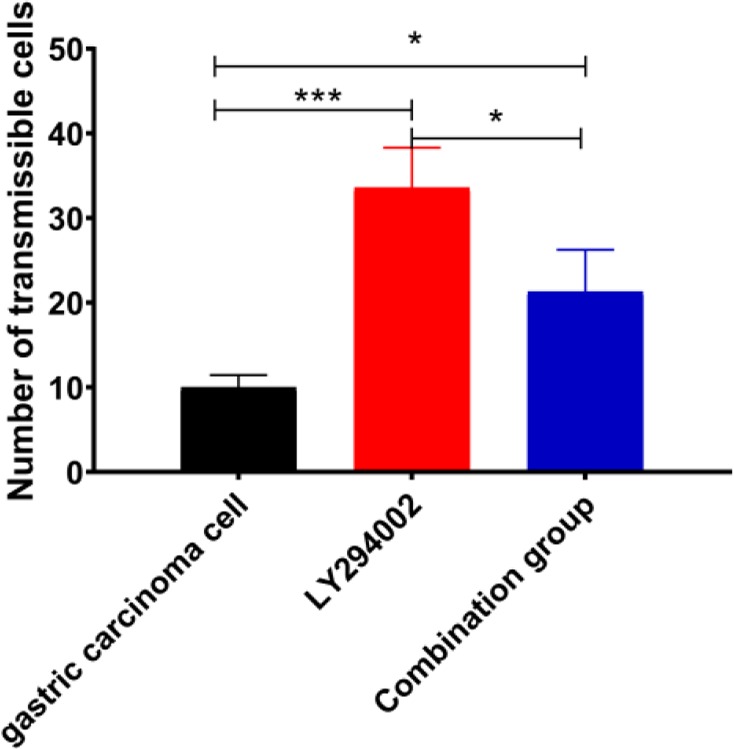
The apoptosis of cells in three groups * represents *P*<0.05; *** represents *P*<0.001

There were significant differences in apoptosis rate between the three groups (*P*=0.001); the apoptosis rate in the LY294002 group (*P*<0.001) and combination group (*P*=0.014) was higher than that in the gastric cancer cell group, and it was lower in the MK-2206 group than in the LY294002 group (*P*=0.010) (Fig. 6).

### The invasion of cells in three groups

**Fig. 7:**
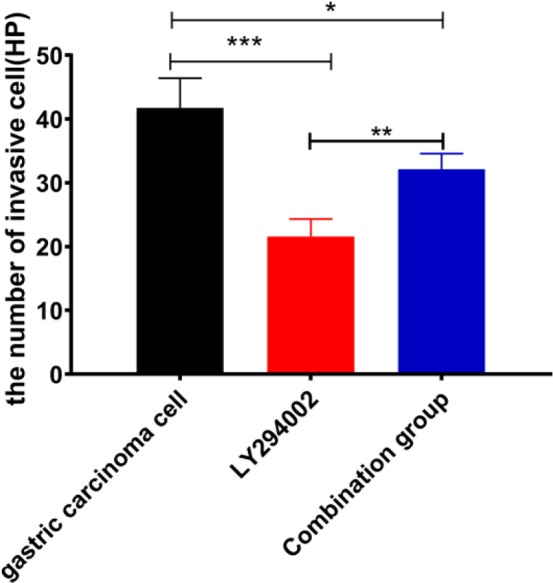
The invasion of cells in three groups * represents *P*<0.05; ^**^ represents *P*<0.01; ^***^ represents *P*<0.001

There were significant differences in the number of transmembrane cells between the three groups (*P*=0.001); the number of transmembrane cells in the LY294002 group (*P*<0.001) and combination group (*P*=0.014) was lower than that in the gastric cancer cell group, and it was higher in the combination group than in the LY294002 group (*P*=0.009) (Fig. 7).

## Discussion

Gastric cancer has high incidence and mortality. Although there are more and more treatments for gastric cancer with the development of medical technology, the therapeutic effect is still not very satisfactory (4, 11). Molecular targeted therapy is a new therapeutic method in recent years. More and more studies have reported that PI3K/AKT signaling pathway can regulate the growth, apoptosis and invasion of tumor cells in malignancies (12, 13). And p-PRAS40-Thr246, a highly specific and stable effector molecule downstream of PI3K/AKT signaling pathway, can predict the sensitivity of prostate cancer cells and triple-negative breast cancer cells to AKT inhibitors, and also affect the prognosis of gastric cancer (9, 10). The relationship between the changes of PI3K/AKT signaling pathway and PRAS40-Thr246 phosphorylation in gastric cancer cells and its effect on the proliferation and apoptosis of gastric cancer cells were analyzed in this study to provide experimental evidence for the molecular therapy of gastric cancer.

Firstly, the levels of PI3K, AKT, p-PI3K, p-AKT, and p-PRAS40-Thr246 in gastric cancer cells and normal gastric mucosal epithelial cells were detected, and it was found that those levels in the gastric cancer cells were higher than those in normal gastric mucosal epithelial cells. Some studies have also reported that the levels of PI3K, AKT, p-PI3K, p-AKT in gastric cancer cells are higher than those in normal gastric epithelial cells (14, 15), which is consistent with our results. There are few reports about PRAS40-Thr246 and gastric cancer. The expression of p-PRAS40-Thr246 in gastric cancer patients with lymph node metastasis, lymph node cell infiltration and vascular infiltration, three highly suggestive markers of tumor cell metastasis and invasion, was significantly higher than that in patients without these symptoms. The higher the expression of p-PRAS40-Thr246, the worse the prognosis of patients with gastric cancer, suggesting that the expression level of p-PRAS40-Thr246 is closely related to the progression of gastric cancer (10).

Increased p-PRAS40-Thr246 expression may increase the risk of progression in breast cancer patients, and may also predict the drug resistance of trastuzumab (16). Phosphorylation of PRAS40-Thr 246 could also mediate abnormal renal lipid metabolism of diabetes mellitus (17) known as a common characteristic of tumor cells (18, 19), but the relationship between p-PRAS40-Thr246 and abnormal renal lipid metabolism of tumor cells has not been confirmed yet. p-PRAS40-Thr246 might play an important role in the occurrence and development of gastric cancer.

After respectively treated with PI3K inhibitor and AKT inhibitor, the level of p-PI3K and p-ATK in gastric cancer cells of the LY294002 group was lower than that in untreated gastric cancer cells; p-AKT level in MK-2206 group was deceased, and the PI3K, AKT levels in both groups had no significant change. Furthermore, the level of p-PRAS40-Thr246 decreased with the intervention. The results of further correlation analysis also showed that there was a positive correlation between p-PRAS40-Thr246 and p-PI3K and p-ATK levels, suggesting that p-PRAS40-Thr246 can also indicate the activation state of PI3K/AKT signaling pathway in gastric cancer cells, thus reflecting the degree of response of PI3K/AKT signaling pathway to inhibitors. The degree of proliferation, invasion and apoptosis of gastric cancer cells after intervention by two inhibitors was measured, and the results showed that the proliferation and invasion of gastric cancer cells treated with inhibitor were significantly lower than those of untreated gastric cancer cells, but the apoptosis level was higher. These results were consistent with previous studies that showed that after inhibiting PI3K/AKT expression, the proliferation of gastric cancer cells was decreased, and the apoptosis level of gastric cancer cells was increased compared with that before intervention (20, 21).

Due to the limitation of experimental conditions, only one gastric cancer cell line was used for verification. The use of both inhibitors also failed to carry out further tests at time and concentration levels. These results will be presented in a series of reports in the future.

## Conclusion

The levels of PI3K/Akt signaling pathway-related proteins and p-PRAS40Thr246 are significantly increased in gastric cancer cells. Inhibition of PI3K/Akt signaling pathway can inhibit the level of p-PRAS40-Thr246 and the ability of cell proliferation and invasion, and also can promote cell apoptosis. p-PRAS40-Thr246 is able to reflect the activation of the PI3K/Akt signaling pathway, reflecting the sensitivity of the PI3K/AKT signaling pathway to inhibitors.

## Ethical considerations

Ethical issues (Including plagiarism, informed consent, misconduct, data fabrication and/or falsification, double publication and/or submission, redundancy, etc.) have been completely observed by the authors.
